# Psychiatric Disease as a Potential Risk Factor for Dementia: A Narrative Review

**DOI:** 10.3390/brainsci14070722

**Published:** 2024-07-18

**Authors:** Dawson W. Hedges, Morgan Chase, Thomas J. Farrer, Shawn D. Gale

**Affiliations:** 1 The Department of Psychology, Brigham Young University, Provo, UT 84602, USA; dawson_hedges@byu.edu; 2 The Neuroscience Center, Brigham Young University, Provo, UT 84602, USA; mchase11@student.byu.edu; 3 Idaho WWAMI Medical Education Program, University of Idaho, Moscow, ID 83844, USA; tfarrer@uidaho.edu

**Keywords:** dementia, incident dementia, neurodegeneration, psychiatric disorders, psychiatric diseases, depression, bipolar disorder, anxiety, post-traumatic stress disorder, obsessive–compulsive disorder, attention-deficit/hyperactivity disorder, schizophrenia, psychotic disorders, autism spectrum disorder, personality disorders, personality traits

## Abstract

Neurodegenerative disease is a major global health problem with 150 million people predicted to have dementia by 2050. Genetic factors, environmental factors, demographics, and some diseases have been associated with dementia. In addition to associations between diseases such as hypertension and cerebrovascular disease and dementia, emerging findings associate some psychiatric disorders with incident dementia. Because of the high and increasing global prevalence of dementia and the high worldwide prevalence of psychiatric disorders, the primary objective of this narrative review was to evaluate published findings that evaluate the association between bipolar disorder, depression, anxiety, post-traumatic stress disorder, obsessive–compulsive disorder, attention-deficit/hyperactivity disorder, autism spectrum disorder, schizophrenia and other psychosis syndromes, and personality disorders and personality traits and incident dementia. Here, we highlight findings indicating possible associations between these psychiatric disorders and subsequent dementia and suggest that some psychiatric disorders may be risk factors for incident dementia. Further research, including more large longitudinal studies and additional meta-analyses, however, is needed to better characterize the associations between psychiatric disorders and incident dementia, to identify possible mechanisms for these putative associations, and to identify risk factors within psychiatric disorders that predispose some people with a psychiatric disorder but not others to subsequent dementia. Additional important questions concern how the treatment of psychiatric disorders might affect the risk of incident dementia.

## 1. Introduction

A major global health problem, neurodegenerative disease can lead to dementia, with approximately 150 million people worldwide predicted to have dementia by 2050 [1]. Dementia is characterized by deficits in cognitive function, including impairment in memory, processing speed, language, attention, and executive function [2]. Multiple different types of dementia exist, including Alzheimer’s disease, the most common type of dementia, which accounts for approximately 60 to 80 percent of dementia cases [3,4]. Other types of neurodegenerative diseases and dementia include vascular dementia, Parkinson’s disease dementia, Lewy-body dementia, frontotemporal dementia, and others [5].

Many factors appear to contribute to dementia. Several genetic variants are strongly associated with Alzheimer’s disease and other dementias, including, for example, variants in the gene for amyloid precursor protein [6]. Nonetheless, these genetic variants account for only a small percentage of the total burden of dementia [7]. However, numerous other risk factors have also been associated with Alzheimer’s disease, including low educational attainment, physical inactivity, smoking, anticholinergic medication use [8], and exposure to some infectious diseases [9,10]. In addition to these risk factors, certain medical conditions are also associated with later dementia, including cardiovascular diseases such as hypertension and cerebrovascular disease [11]. The range of risk factors for dementia suggests that neurodegeneration is not only associated with genetic variants but also numerous environmental risk factors and medical conditions, some of which may be potentially modifiable [8,12].

In addition to associations between medical conditions such as hypertension and dementia [11], accumulating reports have identified potential associations between psychiatric disorders and incident dementia [11], findings suggesting that some psychiatric disorders may be risk factors for dementia [11]. In a study across 30 years including 1,711,386 people in New Zealand, for instance, mental disorders were associated with subsequent dementia (risk ratio: 4.24; 95 percent confidence interval: 4.07 to 4.42). Moreover, in patients with dementia, those with a history of mental disorder had an onset of dementia on average 5.8 years earlier than those people with dementia but without a history of mental disorder [13]. Bipolar disorder [14], depressive disorders [15], post-traumatic stress disorder [16], schizophrenia [17], attention-deficit/hyperactivity disorder [18], and obsessive–compulsive disorder [19] are among the psychiatric disorders that have been associated with incident dementia. The goal of this narrative review is to evaluate the hypothesis that a variety of psychiatric disorders could be risk factors for incident dementia.

## 2. Associations between Psychiatric Disorders and Incident Dementia

### 2.1. Bipolar Disorder

Bipolar disorder is characterized by alternating periods of mania and depression and affects approximately two percent of the global population [20]. A variety of studies have associated bipolar disorder with subsequent dementia [21], including frontotemporal dementia [22] and Parkinson’s disease [23]. Individuals diagnosed with bipolar disorder may have up to 3 times the risk of developing dementia than individuals without bipolar disorder [24]. A 2017 systematic review and meta-analysis based on six primary studies found that bipolar disorder increased the risk for subsequent dementia (odds ratio: 2.36; 95-perecent confidence interval: 1.36 to 4.09). Based on their findings, the authors of this meta-analysis concluded that at least in a subgroup of patients, bipolar disorder could be a progressive disease associated with later dementia [25]. Another systematic review based on longitudinal studies that was unable to quantitatively pool results found that bipolar disorder was associated with subsequent dementia [26].

One category of dementia that has implications with bipolar disorder is frontotemporal dementia. Indeed, bipolar disorder has been associated with the behavioral variant of frontotemporal dementia, which is associated with personality and emotional changes that are not present in other types of frontotemporal dementia [27]. Mendez et al. [27] noted a complex relationship between bipolar disorder and behavioral-variant frontotemporal dementia. Many symptoms of bipolar disorder and behavioral-variant frontotemporal dementia overlap, but a further examination of the psychiatric history of the patients led Mendez and colleagues to conclude that some cases of bipolar disorder could be a prodrome of behavioral-variant frontotemporal dementia [27]. One systematic review found that despite biological and neuropsychological differences between the two conditions, 10.2 to 11.6 percent of patients with frontotemporal dementia had a history of bipolar disorder [22], higher than the two percent global prevalence of bipolar disorder l [20]. Based on data from case reports, the mean transition time from the diagnosis of bipolar disorder to the onset of frontotemporal dementia was 24.5 years. Moreover, bipolar disorder and frontotemporal dementia share genetic risk variants. These findings suggest that some cases of bipolar disorder could be at risk for subsequent frontotemporal dementia [22].

In their review, Serafini et al. [28] argue that the available data including molecular, neurocognitive, neuroimaging findings suggest that at least some cases of bipolar disorder can lead to some degree of neurodegeneration. Serafini et al. [28] further argue for additional study to better characterize cognitive phenotypes of bipolar disorder to identify those with potential for neurodegeneration. In addition, Serafini et al. [28] note findings about the neuroprotective properties of lithium and how it could prevent the neurodegenerative aspects of bipolar disorder.

In addition to associations between bipolar disorder and dementia, including frontotemporal dementia, bipolar disorder has also been associated with Parkinson’s disease. In a study based on data from the Taiwan National Health Insurance Research database, Huang et al. [23] found that bipolar disorder was associated with an increased risk of incident Parkinson’s disease, with a hazard ratio of 6.78 (95 percent confidence interval: 5.74 to 8.02). In addition, Huang et al. [23] found that both manic and depressive episodes were associated with an increased risk of subsequent Parkinson’s disease. Further, a 2020 meta-analysis of seven studies that included 4,374,211 participants found that bipolar disorder increased the risk of developing later Parkinson’s disease with an odds ratio of 3.35 (95 percent confidence interval: 2.00 to 5.60) [29]. Noting involvement of dopamine circuitry in both bipolar disorder and Parkinson’s disease, Geelhand de Merxem et al. [30] in their review reported that bipolar disorder appears to increase the risk of later Parkinson’s disease but cautioned, however, that the association could be due to antipsychotic medication and anticonvulsant use in patients with bipolar disorder.

In a machine learning predictive dementia model using administrative claims data from 125 million patients, the diagnostic codes most strongly associated with incident dementia were memory loss, Parkinson’s disease, mild cognitive impairment (MCI), and bipolar disorder [14].

Research suggests that bipolar disorder exacerbates functional and cognitive decline and could be a risk factor for subsequent dementia, possibly through an association with accelerated brain aging, particularly in older adults, and structural changes [24,31]. Many individuals with bipolar disorder have increased ventricular volume, which is thought to be a predictor of changes to white matter and gray matter. These factors together suggest that bipolar disorder might be associated with accelerated brain aging [32], although not all findings support an association between bipolar disorder and accelerated brain aging [33,34]. Additional changes in brain structure over time, including changes in prefrontal cortex, temporal cortex, right fusiform gyrus, right parahippocampus, and the amygdala, and the number of episodes of mania appear to be related to decreased gray matter in prefrontal regions [24,31].

As these findings associating bipolar disorder with subsequent dementia and neurodegenerative disease suggest, bipolar disorder has been associated with incident neurodegenerative disease, including frontotemporal dementia and Parkinson’s disease. More research, though, is indicated to verify and characterize these associations and to identify subtypes of bipolar disorder prone to the development of subsequent neurodegeneration. An important question is whether lithium treatment of bipolar disorder affects the risk of subsequent neurodegeneration in patients with bipolar disorder.

### 2.2. Depression

Associated with depressed mood, anhedonia, sleep and appetite changes, and decreased energy [35], depression has a lifetime prevalence of approximately 14.6 percent in high-income countries and 11.1 percent in low- to middle-income countries [36], with a 12-month prevalence of approximately 3.7 percent [37]. It is estimated that more than 264 million people suffer from major depressive disorder worldwide, with recurrent episodes estimated as being as high as 75 to 90 percent [38]. Depression and dementia can be comorbid [39,40], although the exact nature of the relationship between depression and dementia is unclear. Depression could be a risk factor for dementia, or depression could be a prodrome for dementia [39,40].

A variety of research has investigated the association between depression and dementia. In participants from the Framingham Heart Study, depression increased the risk of incident dementia over a 17-year follow-up, even after controlling for other risk factors for dementia including apolipoprotein E status [41].

Several systematic reviews and meta-analyses have also shown an association between depression and an increased risk of developing dementia later in life [42,43,44]. In their 2020 meta-analysis of eight longitudinal studies, Santabárbara and colleagues [44] found that depression was associated with an elevated risk of dementia (relative risk: 1.63; 95 percent confidence interval: 1.30 to 2.04). In addition, this meta-analysis found a population-attributable risk of 9.0 percent (95 percent confidence interval: 4.5 percent to 14.1 percent), indicating that if depression is indeed a cause of dementia, then 9 percent of all cases of dementia would not occur if depression were eliminated [44].

In order to try to understand the nature of the association between depression and dementia, depression at various stages of life and subsequent dementia have been investigated, as the time of the onset of depression could be important [39]. Depression in early life appears associated with incident dementia [45], with possibly as much as a two-fold increase in dementia risk [39]. A study of 595,828 Danish men from the Danish Conscription Database found that depression before late midlife increased the risk of later dementia in adjusted models [46]. Depression in late life has also been associated with dementia, but it is uncertain whether depression is a risk factor for dementia, a prodrome of dementia, or the result of dementia [39,45]. Some complications in understanding this relationship are the comorbidity between depression and dementia and symptom overlap, which can complicate understanding the association between depression and dementia [47]. In their 2022 review, Kawakami et al. suggested that there could be shared pathophysiology between late-life depression and dementia [47].

A study of 356,052 participants from the UK Biobank found that depression was associated with young-onset dementia, defined as dementia onset before age 65 years, with a hazard ratio of 3.25 (95 percent confidence interval: 2.08 to 5.09). Notably, among the 15 variables associated with an elevated risk of young-onset dementia in this study in the fully adjusted models (lower formal education, social isolation, lack of use of alcohol, alcohol use disorder, diabetes, hearing impairment, heart disease, high C-reactive protein, lower hand-grip strength, orthostatic hypotension, stroke, two apolipoprotein ε4 alleles, and vitamin D deficiency), only the hazard ratio for orthostatic hypotension was higher than the hazard ratio for depression [15].

In their study using machine learning based on 125 million patients that found bipolar disorder to be the fourth most strongly associated diagnosis with incident dementia, Nori et al. [14] also found that depressive disorder not otherwise specified was the seventh most strongly associated diagnosis with incident dementia. In this same study, the antidepressants venlafaxine, duloxetine, sertraline, and citalopram were four of the five most strongly associated pharmacy codes associated with incident dementia, possibly providing further support for an association between depression and incident dementia.

A 2022 meta-analysis of longitudinal studies evaluating the association between depression and dementia found that depression was associated with all-cause dementia (relative risk: 1.96; 95 percent confidence interval: 1.59 to 2.43), Alzheimer’s disease (relative risk: 1.90; 95 percent confidence interval: 1.52 to 2.38), and despite mixed findings in primary studies, vascular dementia (relative risk: 2.71; 95 percent confidence interval: 2.48 to 2.97). The authors also calculated a population-attributable fraction of 3.07 percent, assuming that the observed association between depression and dementia is causal [26].

Liu et al. [48] in an 11-year study using the National Health Insurance Research Database from Taiwan found a bidirectional relationship between depression and dementia, with a higher incidence density of dementia in patients with major depression than in patients without major depression (adjusted hazard ratio: 2.71) and a higher incidence density of depression in patients with dementia than in patients without dementia (adjusted hazard ratio: 2.47). While major depressive disorder could be an early manifestation of dementia or part of a dementia prodrome, Liu et al. [48] suggested that their results were consistent with major depressive disorder being a risk factor for subsequent dementia. Further assessing their findings, Liu et al. [48] speculated that neurophysiological abnormalities associated with major depressive disorder could affect the risk for dementia.

In cognitively intact older adults, subclinical depressive symptoms were associated with decreased gray matter in the hippocampus and with glucose hypometabolisms in a variety of regions including the dorsolateral prefrontal cortex, hippocampus, and insula, although there was no association between the subclinical symptoms of depression and amyloid. The abnormalities related to subclinical depression in this frontolimbic network are also found in Alzheimer’s disease [49], suggesting possible anatomic and functional links between depressive symptoms and neurodegenerative disease.

Using machine learning on data from National Health Insurance Research Database in Taiwan to identify disease pathways leading to dementia, Huang et al. [11] found that, in addition to associations with cardiovascular and cerebrovascular disease, decreased mobility, and infectious diseases, there are also associations between depression and anxiety with dementia occurrence.

Findings based on 109,140 female veterans with a mean age of 68.5 years in the Veterans Health Administration found that depression increased the chances of incident dementia over a four-year follow-up by a factor of 1.67. In this study, women who had a combination of depression, post-traumatic stress disorder (PTSD), or traumatic brain injury were 2.2 times more likely to develop dementia as women without depression, PTSD, or traumatic brain injury [50].

Finally, whether successful treatment of depression may reduce the risk of dementia is unclear. However, at least one meta-analytic study based on four primary studies found that antidepressant use was not protective but associated with an increased risk of subsequent mild cognitive impairment and dementia [43]. However, additional studies are needed to better characterize how treatment for depression affects the association between depression and incident dementia.

As a group, findings from studies including meta-analyses and large longitudinal studies indicate associations between depression and subsequent dementia. However, the relationship between depression and later dementia is unclear, as depression could be a prodrome to, an early sign of, or a risk factor for dementia. All three types of relationships could be involved in the associations between depression and dementia. Further, research investigating risk factors in depression for the later occurrence of dementia is needed. In addition, few available studies address how antidepressant treatment alters the association between depression and subsequent dementia. Clearly, more research is needed to determine if certain types of antidepressant treatment or treatment intervention at critical times would affect the relationships between depression and dementia.

### 2.3. Anxiety

Although there are several different anxiety disorders including generalized anxiety, panic disorder, and social anxiety disorder, they are all characterized by inappropriately elevated anxiety, even though the precipitant of the anxiety may differ between anxiety disorders [51]. As a group, anxiety disorders are common, with a lifetime prevalence of approximately 34 percent [51]. Like several other psychiatric disorders, anxiety has been associated with an increased risk of incident dementia.

In a meta-analysis investing whether anxiety affects progression from mild cognitive impairment to dementia, Li and Li [52] found that anxiety in people with mild cognitive impairment increased the risk of dementia compared to people with mild cognitive impairment who did not have anxiety (hazard ratio: 1.18; 95 percent confidence interval: 1.07 to 1.31), although the risk ratio in this meta-analysis showed no differences between the groups with and without anxiety. The risk ratio did not adjust for confounding variables such as education and age. Additionally, the hazard ratio accounted for follow-up time for assessing dementia progression, which Li and Li [52] claim to be more appropriate for assessing the association between anxiety and the progression from mild cognitive impairment to dementia [52], despite the contradictory results from the hazard and risk ratios. In addition to the results from the Huang et al. [11] study previously mentioned that showed an association between anxiety and subsequent dementia, a 2019 meta-analysis and systematic review based on prospective studies found that anxiety was associated with incident dementia (risk ratio: 1.29; 95 percent confidence interval: 1.01 to 1.66) [53]. In their meta-analysis of seven longitudinal studies, in contrast, Stafford et al. [26] found inconsistent findings among the primary studies with no pooled association between anxiety and incident dementia (relative risk; 1.18; 95 percent confidence interval: 0.96 to 1.45). Stafford et al. [26] calculated a population-attributable fraction of 1.05 percent for anxiety and dementia.

In another study of anxiety—which included anxiety disorder not otherwise specified, generalized anxiety disorder, panic disorder, and social anxiety disorder—anxiety in older adults was associated with incident dementia (hazard ratio: 1.19; 95 percent confidence interval: 1.06 to 1.33). The use of benzodiazepines did not appear to affect this association [54]. In another longitudinal 2023 study of what the authors referred to as clinically significant anxiety in a community-based sample of participants aged 65 years or older, Gracia-García et al. [55] found that in adjusted models including adjustment for depression, anxiety was associated with later Alzheimer’s disease (sub-distribution hazard ratio: 2.82; 95 percent confidence interval: 1.21 to 6.58).

Anxiety also has been associated with Parkinson’s disease. Among 35,815 male participants in a 12-year prospective study, anxiety was associated with incident Parkinson’s disease [56]. Using data from the Taiwan National Health Insurance Research Database, Lin et al. [57] found an association between anxiety and incident Parkinson’s disease over a mean follow-up of 5.5 years, with more severe anxiety more strongly associated with Parkinson’s disease. Based on clinical data involving 987,691 patients from the United Kingdom, new-onset anxiety at or after age 50 years was associated with double the risk of subsequent Parkinson’s disease. Since this study involved new-onset anxiety in patients aged 50 years or older, the anxiety could have been a prodrome to and not necessarily a risk factor for Parkinson’s disease [58].

Despite the high prevalence of anxiety disorders [51], comparatively few studies have investigated associations between anxiety disorders and incident dementia. However, outcomes from the available evidence are mixed but do suggest the possibility that anxiety disorders could be associated with subsequent dementia, although additional research investigating this association is needed, as is work aimed at identifying the factors in anxiety disorder that could increase the risk for subsequent dementia.

### 2.4. Post-Traumatic Stress Disorder

With a prevalence of approximately 6 percent, post-traumatic stress disorder (PTSD) is characterized by the avoidance of reminders of the trauma, hypervigilance, and re-experiencing memories of the trauma [59]. Emerging findings suggest that post-traumatic stress disorder (PTSD) may be a risk factor for subsequent dementia.

Logue et al. found an association between PTSD and Alzheimer’s disease and related dementias [60]. In this regard, Yaffe et al. [16] found that male veterans with PTSD were twice as likely as male veterans without PTSD to develop dementia, findings similar to those from a more recent study showing that female veterans with PTSD were 1.78 times more likely than women without PTSD to develop dementia during a follow-up of an average of four years [50]. In this study, women with more than one diagnosis of traumatic brain injury, depression, and PTSD were 2.15 times more likely to develop dementia than women without one of these diagnoses. Similarly, Meziab et al. [61] found a more than 75 percent increase in the risk of dementia for veterans with a diagnosis of PTSD and veterans with both PTSD and prisoner-of-war status had more than double the risk of dementia. Moreover, a meta-analysis [62] found that civilian adults over 60 years with war-related PTSD had worse cognitive function on a range of neurocognitive tasks compared to civilians exposed to war but without PTSD and to healthy controls with small to moderate effect sizes. These findings suggest that in older adults, civilian exposure to war with the development of PTSD could also be a risk factor for subsequent dementia, although additional research addressing whether cognitive impairment in elderly people with a history of civilian war exposure is also associated with dementia is needed.

More recently, Neale and colleagues [63] found that PTSD was a risk factor for dementia. In their analysis, PTSD was strongly linked with higher subjective cognitive concern scores, which were also found to be an indicator of future dementia diagnoses and a potential prodrome state of dementia [63].

A study based on data from the Taiwan National Health Insurance Database found that PTSD was associated with the later development of dementia (hazard ratio: 3.46; 95 percent confidence interval: 1.72 to 6.96) [64]. While unable to conduct a pooled quantitative analysis due to too few studies, Stafford et al. [26] reported that all three groups included in their systematic review showed an association between PTSD and subsequent dementia.

The mechanism by which PTSD may act as a risk factor for dementia is not well understood. Miller et al. [65] studied biological indicators of dementia and found an interaction between PTSD and Factor TN, as well as between PTSD and glial fibrillary acidic protein, but not with APOE e4 or ATN biomarkers (amyloid-beta deposition (A), pathologic tau (T), neurodegeneration (N)).

Taken as a whole, the available research suggests that PTSD could be a risk factor for the later development of dementia, although the relatively few available studies indicate that more research evaluating this association and delineating factors in PTSD associated with incident dementia is needed, as is research identifying risk factors within PTSD for the development of subsequent dementia.

### 2.5. Obsessive–Compulsive Disorder

Among the psychiatric disorders that have been associated with subsequent dementia is obsessive–compulsive disorder [66,67]. Obsessive–compulsive disorder is part of a spectrum of disorders called obsessive–compulsive and related disorders [68]. This disorder is characterized by thoughts, impulses, urges (obsessions) and/or behaviors, or mental routines (compulsions) that are repetitive and often unwanted and beyond the control of the individual, causing significant distress or impaired social and/or occupational functioning [68]. The lifetime prevalence of obsessive–compulsive disorder is thought to be 2 to 3 percent. Obsessive–compulsive disorder is highly comorbid with other psychiatric disorders, such as anxiety disorders and affective disorders [68].

Several case reports have suggested a possible association between obsessive–compulsive disorder and incident dementia. In one, a 57-year-old woman with a five-year history of significant obsessive–compulsive symptoms was evaluated and treated, although the symptoms of obsessive–compulsive disorder did not respond well to treatment. At age 64 years, the patient was diagnosed with dementia. Test results, including neuroimaging, were the most consistent with a diagnosis of Alzheimer’s disease [69]. In another case study, a 75-year-old woman with a history of obsessive–compulsive disorder since adolescence also demonstrated Alzheimer’s type dementia. These authors suggested the possibility that obsessive–compulsive disorder may be a risk factor for Alzheimer’s disease [67]. In another case report, late-onset obsessive–compulsive disorder was associated with a diagnosis of frontotemporal dementia 10 years later [70]. Similarly, another case study reported a patient who had late-onset obsessive–compulsive disorder at age 57 years and who was subsequently diagnosed with primary lateral sclerosis and frontotemporal dementia at age 64 years [71]. In describing the case of a 46-year-old with late-onset obsessive–compulsive disorder type symptoms, Ducharme et al. [72] discussed the difficulty and importance of differential diagnosis between the behavioral variant of frontotemporal dementia from primary psychiatric conditions. Indeed, regarding associations between obsessive–compulsive disorder and the development of frontotemporal dementia, it will be particularly important to differentiate late-onset obsessive–compulsive disorder, which could be an early expression of behavioral variant frontotemporal dementia rather than a causative condition, from those with obsessive–compulsive disorder onset at a typical age who later develop frontotemporal dementia. A case study by Ruggeri and colleagues [73] suggested the possibility of late-onset obsessive–compulsive disorder being a precursor to a behavioral variant of Alzheimer’s Disease. Similarly, Frileux et al. [74] reported a case study with two patients that developed late-onset obsessive–compulsive disorder and later developed Lewy-body dementia. These three case studies indicate the need for more research on the relationship between late-onset obsessive–compulsive disorder and different forms of dementia to determine if the association seen in these cases is indicative of late-onset obsessive–compulsive disorder leading to dementia or if the symptoms of late-onset obsessive–compulsive disorder are symptoms of dementia.

In a study of 39 people diagnosed with Alzheimer’s disease and 30 healthy controls, Dondu et al. [66] found a higher lifetime prevalence of obsessive–compulsive symptoms and current symptoms in those with Alzheimer’s disease. Finally, using data from the Taiwan National Health Insurance Database, Chen et al. [19] found that obsessive–compulsive disorder was associated with subsequent dementia of any type (hazard ratio: 4.28; 95 percent confidence interval: 2.96 to 6.21), Alzheimer’s disease (hazard ratio: 4.04:1.55 to 10.54), and vascular dementia (hazard ratio: 3.95:1.70 to 9.18).

While the case reports linking late-onset obsessive–compulsive disorder with dementia could suggest that the obsessive–compulsive features in these patients could have been a prodrome or early manifestation of the dementia, the findings of Chen et al. [19] based on data from the Taiwan National Health Insurance Database more strongly suggest that obsessive–compulsive disorder could be a risk factor for incident dementia. Moreover, the hazard ratio in the study of Chen et al. [19] was 4.28 for all-cause dementia. For comparison, a reported hazard ratio for the association between one copy of the apolipoprotein epsilon E4 allele was 2.19 (95 percent confidence interval: 1.73 to 2.77) and for two copies was 5.97 (95 percent confidence interval: 3.85 to 9.28) [75].

Liu and colleagues [76] used data from the UK Biobank and performed a Mendelian randomization analysis to study the association between Alzheimer’s disease and multiple psychiatric disorders, including obsessive–compulsive disorder. Their results indicate that there may be an association between obsessive–compulsive disorder and Alzheimer’s disease, although the mechanism for this association needs additional investigation [76]. Sair and Sair [77] found that a previous diagnosis of obsessive–compulsive disorder had a significant association with mild cognitive impairment, which was associated with an increased risk of developing dementia [78].

While comparatively few studies have investigated the relationships between obsessive–compulsive disorder and dementia, the hazard ratio for the association between obsessive–compulsive disorder and incident dementia reported by Chen et al. [19] underscores the need for additional research investigating the association between obsessive–compulsive disorder and incident dementia.

### 2.6. Attention-Deficit/Hyperactivity Disorder

Several studies have investigated the possible association between attention-deficit/hyperactivity disorder (ADHD) and later dementia [79]. ADHD is characterized by deficits in attention and by hyperactivity [80]. According to the results of a meta-analysis, the prevalence of ADHD in children ages 3 to 12 years is 7.8 percent. In adolescents between the ages of 12 and 18 years, the prevalence is 5.6 percent [81]. In adults, the prevalence of ADHD is approximately 2 to 4 percent [18].

In their review of eight published studies investigating the association between ADHD and incident dementia, Becker et al. [18] concluded that the eight reviewed studies provide some support for an association between ADHD and incident dementia, particularly for Lewy-body-associated dementia, but noted further that methodological limitations require additional research to better characterize this association. More recently, Golimstok et al. [82] completed a prospective cohort study and found that while a diagnosis of ADHD at baseline was associated with an increased risk for the development of mild cognitive impairment, a non-amnestic subtype, Alzheimer’s dementia, and vascular dementia, the strongest association was with Lewy-body-associated dementia. In contrast, a 2023 systematic review found that ADHD was associated with all-cause dementia and other types of dementia, specifically vascular dementia, indicating that individuals with ADHD were 6 times more likely to develop vascular dementia than individuals without ADHD [79]. Additionally, Becker and colleagues found an association between ADHD and dementia with Lewy bodies (incidence rate ratio 1.06) and between ADHD and Parkinson’s disease [79]. The authors hypothesize that this association between ADHD and dementia with Lewy bodies and between ADHD and Parkinson’s disease is linked by pathology in the dopaminergic system [79]. However, there seemed to be lower risk of individuals with ADHD developing mild cognitive impairment, and all-cause dementia had variable risk (adjusted hazard ratio range: 0.98–4.01) [79].

Although some findings support a possible association between ADHD and Lewy-body dementia [18], one study found no evidence that nine genetic variants possibly associated with attention-deficit/hyperactivity disorder are involved in Parkinson’s disease [83]. In contrast, polygenic risk scores for ADHD in cognitively healthy people without ADHD were associated with incident dementia and pathological markers of Alzheimer’s disease [84]. One gene that may be a link between Alzheimer’s disease and ADHD is the SORCS2 gene. Single-nucleotide polymorphisms in this gene seem to play a role in some of the symptoms associated with ADHD, as well as being involved with processing amyloid precursor protein into amyloid-beta [85]. However, not all studies support this hypothesis. A study by Pagoni et al. [86], did not find a causal link between ADHD and Alzheimer’s disease and did not find evidence of ADHD being a risk factor for Alzheimer’s disease.

Other studies have also noted an increased risk for dementia in those with a diagnosis of ADHD. One prospective cohort study, which did not differentiate between dementia subtypes, found that adult ADHD was associated with an increased risk of subsequent dementia (hazard ratio: 2.77; 95 percent confidence interval: 2.11 to 3.63) [87]. Another large cohort study that also included all types of dementia, but with ADHD diagnosed at any age, found an increased risk for dementia (hazard ratio: 2.92; 95 percent confidence interval: 2.40 to 3.57) and mild cognitive impairment (hazard ratio: 6.21; 95 percent confidence interval: 5.25 to 7.35), which was found to be 58 percent higher in men than in women [88]. Notably, the hazard ratios decreased for dementia and mild cognitive impairment, respectively, to 1.62 and 2.54, when adjusted for other psychiatric disorders, a finding that also suggests the possibility that interactions between psychiatric disorders could influence the association between ADHD and later dementia, and indeed between other psychiatric disorders and incident dementia.

Although the mechanisms for the possible associations between ADHD and dementia are unclear, some have suggested that ADHD may increase susceptibility to the negative consequences of brain aging [87]. Others have highlighted the pathophysiological overlap between ADHD and Alzheimer’s disease, including wingless-INT/mammalian target of rapamycin signaling, as needing further research [89].

While additional research investigating the association between ADHD and incident dementia are needed, the available findings indicate that ADHD may be a risk factor for the development of subsequent dementia, including whether any particular factors confer a risk of or protection against later dementia in people with ADHD.

### 2.7. Autism Spectrum Disorder

Autism spectrum disorder is a neurodevelopmental disorder that develops during infancy, with symptoms including difficulty with socialization and communication and repetitive behaviors [90]. Despite these common symptoms, the presentation of autism spectrum disorder is quite heterogeneous, including various levels of cognitive impairment [91]. The estimated global prevalence of autism spectrum disorder is approximately 1 percent, although there is considerable demographic and geographic variation in prevalence [92].

While few studies have investigated the associations between autism spectrum disorder and subsequent neurodegenerative disease, emerging findings suggest that autism spectrum disorder could be a risk factor for subsequent dementia, although the associations between autism spectrum disorder and incident dementia remain unclear [93,94]. In 2014, Oberman and Pascual-Leone suggested that Asperger’s syndrome, a part of the autism spectrum, is protective against dementia because of synaptic hyperplasticity [95]. Additionally, they investigated the prevalence of individuals having diagnoses of both autism spectrum disorder and Alzheimer’s disease and found it to be lower than predicted. In looking at cognitive decline, some studies have found no difference between adults with autism spectrum disorder and those without [96,97]. In contrast, Croen et al. [98] found that adults with autism spectrum disorder were 4 times more likely to have dementia than those without autism spectrum disorder (odds ratio: 4.40; 99 percent confidence interval: 2.50–7.71). Similarly, Vivanti et al. [99] found no evidence of autism spectrum disorder being protective against dementia but instead found autism spectrum disorder to be a risk factor for dementia. Using an insurance database (Medicaid), they identified persons diagnosed with autism spectrum disorder to determine the incidence and prevalence of early-onset dementia (diagnosis of dementia before age 65 years) over a five-year period [99]. The prevalence of early-onset dementia in those with autism spectrum disorder without intellectual disability was 4.04 percent and 5.22 percent in those with autism spectrum disorder who were also diagnosed with intellectual disability. Overall, those diagnosed with autism spectrum disorder were approximately 2.6 times as likely to be diagnosed with early-onset dementia compared to the general population [99]. The incidence was higher in those with autism spectrum disorder and intellectual disability (adjusted hazard ratio: 2.89; 95 percent confidence interval: 2.62 to 3.17) compared to those with ASD only (adjusted hazard ratio: 1.96; 95 percent confidence interval: 1.69 to 2.28). The risk of incident dementia for those identified as having an intellectual disability was 3.01 (95 percent confidence interval: 2.87 to 3.15). It is not necessarily surprising that intellectual disability is a risk factor for dementia as it has long been known that Down syndrome, a common cause of intellectual disability, has been strongly linked to Alzheimer’s disease, with estimated prevalences of AD among those diagnosed with Down syndrome of 13 percent at age 55 years and as high as 80 percent for those older than 60 years [100]. Notably, as there appear to be common pathologies shared between Down syndrome and Alzheimer’s disease, there is also evidence for pathological commonalities between autism spectrum disorder and Alzheimer’s disease [101]. In addition, a variety of other genetic neurodevelopmental disorders including fragile X syndrome, Prader-Willi syndrome, and Mbd5-associated neurodevelopmental disorder are also associated with early-onset dementia [102].

Findings from studies evaluating the association between autism spectrum disorder and incident dementia are mixed, indicating the need for additional research into the association between autism spectrum disorder and dementia. More research into the different subtypes of autism spectrum disorder and dementia is needed to better characterize the relationship between autism spectrum disorder and incident dementia [95,99].

### 2.8. Schizophrenia

A complex neuropsychiatric disease with a high illness burden, schizophrenia has an estimated global point prevalence of 0.28 percent [103]. Much has been written about brain morphological abnormalities in people with schizophrenia, with extensive evidence of abnormal dendritic spines, decreased cortical gray matter volume, increased ventricle size, and white matter abnormalities [104,105,106]. van Erp et al. [107] also demonstrated that those with schizophrenia display reduced volume in the hippocampus, thalamus, and accumbens, and multiple studies suggest that brain structural and functional deficits are associated with the symptom profile and cognitive dysfunction that characterize schizophrenia [108,109]. These imaging studies raise the concern that those with schizophrenia may have less resilience (i.e., less cognitive reserve) through aging and may have an increased risk of dementia later in life.

Several studies have explored the risk of dementia among those with schizophrenia. In a large longitudinal cohort study of 2.8 million, Ribe et al. [110] followed individuals over 18 years to monitor incident dementia and examined dementia risk with incident rate ratios (IRRs). This study found that those with schizophrenia were twice as likely to develop dementia (IRR: 2.13; 95 percent confidence interval: 2.00 to 2.27); further, this effect remained stable after controlling for multiple comorbidities.

Using a random sample of over 8 million Medicare beneficiaries, Stroup et al. [17] explored dementia risk in those diagnosed with or without schizophrenia. In the total sample, over 74,000 individuals were diagnosed with schizophrenia, and at age 66, the prevalence of a dementia diagnosis in the schizophrenia group was 27.9 percent relative to 1.3 percent of those without schizophrenia. The study also suggested there may be an effect of age, with further risk later in life. Specifically, by age 80 years, the prevalence of dementia among those with schizophrenia was 70.2 percent compared to 11.3 percent for those without schizophrenia.

Cognitive deficits in schizophrenia and dementia are strikingly similar [111], and older adults with schizophrenia who also carried a greater symptom severity and longer institutionalization had the greatest risk of cognitive decline [112]. In a 25-year longitudinal study, Jonas et al. [113] followed 428 individuals (212 with schizophrenia and 216 with other psychotic conditions). The authors found that those with schizophrenia not only demonstrated cognitive decline up to 14 years before illness onset but also displayed a sharper decline in cognition over the study period, losing an average of 16 IQ points during the study period and meeting the criteria for mild cognitive impairment by age 55. Similarly, Friedman et al. [114] found that older adults with schizophrenia demonstrated greater declines in cognition over a 6-year follow-up period relative to younger adults with schizophrenia. Additionally, this age-related sharp decline was more pronounced in those with schizophrenia than in people with Alzheimer’s disease or in healthy normal-aging controls.

Finally, a meta-analysis of dementia risk in schizophrenia [115] demonstrated a relative risk ratio (RR) of 2.29 (95 percent confidence interval: 1.35 to 3.88). These findings are supported by a recent study by Liou et al. [116], which also demonstrated a greater risk of Alzheimer’s disease for those with schizophrenia (hazard ratio: 4.5; 95 percent confidence interval: 2.84 to 7.13) and a greater risk of vascular dementia (hazard ratio: 4.55; 95 percent confidence interval: 3.14 to 6.59). A 2022 meta-analysis found that psychotic disorders were associated with subsequent dementia (relative risk: 2.19; 95 percent confidence interval: 1.44 to 3.31; population-attributable fraction: 0.083 percent) [26]. Across multiple studies employing various methods, there is clear converging evidence that schizophrenia is associated with an increased risk of dementia.

While there seems to be consistent evidence that schizophrenia is associated with an increased risk of dementia, there are multiple possible explanations for this risk and mixed literature on the underlying causal mechanisms. First, multiple studies seem to suggest that the risk is not associated with the same putative pathological markers observed in Alzheimer’s disease [117]. While these studies suggest that schizophrenia is not inordinately prone to neuropathology associated with Alzheimer’s diseases or other pathological substrates (such as α-synuclein) [118], some studies have postulated that schizophrenia and certain dementia phenotypes share underlying genetic causes [119,120]. Other potential mechanisms underlying associations between schizophrenia and subsequent dementia include low cognitive reserve [121] and cardiovascular disease, which may be more frequent among individuals with schizophrenia [122], and which are associated with a greater prevalence of cerebral vascular disease and subsequent dementia [123].

### 2.9. Other Psychosis Syndromes

It is well described that patients with dementia often have symptoms of psychosis. For example, psychotic symptoms were observed in 31 percent of dementia patients in one sample [124]. In their narrative review, Fischer and Agüera-Ortiz [125] argue that psychosis is common in what they call prodromal dementia and further note findings showing that schizophrenia is associated with an elevated risk of subsequent dementia. An additional finding from the study by Nori et al. [14] based on an administrative claims database was that the unspecified psychosis code was the fifth most strongly related diagnostic code related to incident dementia, providing additional data for an association between psychosis, although not necessarily schizophrenia, and incident dementia. Similarly, a population-based longitudinal cohort study found that of those adults diagnosed late in life (age 60 years or older) with a non-affective psychotic disorder, including schizophrenia, 8 percent were subsequently diagnosed with dementia [126]. A systematic review of 17 studies published between 1987 and 2020 on the association between psychosis and subsequent dementia suggested the need for additional studies, particularly those with longitudinal designs, clearer diagnostic criteria for late-onset schizophrenia-like psychosis, the inclusion of neuroimaging data or other measures of neuropathology, and improved study quality including less heterogeneity in measures of outcome and included populations [127]. Yang et al. [127] also suggested that there may not be a substantial difference between early-onset and late-onset schizophrenia and late-onset schizophrenia-like psychosis in terms of cognitive decline, and that there is a need for direct comparisons of neuropathology between these conditions and dementia.

In summary, increasingly robust research findings associate schizophrenia with an increased risk for incident dementia. In addition to the need for additional research to better characterize this association, further research into factors conferring risk for dementia in people with schizophrenia and how treatment might affect these associations is needed.

### 2.10. Personality Disorders and Personality Traits

Although there are several different personality disorders, long-term traits such as behavioral inflexibility, impaired self-regulation of emotions and impulses, and maladaptive interpersonal skills and relationships that impair overall function characterize personality disorders as a group [128,129]. Personality disorders affect approximately 10.7 to 14.5 percent of adults above the age of 50 years, and the prevalence in those with dementia has been estimated at 25.8 percent [130]. Some evidence has suggested associations between personality disorders [131] and personality traits [132] and subsequent dementia.

In a study of women with Alzheimer’s disease compared to a control group of women without Alzheimer’s disease, assessments of premorbid personality function by close relatives of the participants showed that the group with Alzheimer’s disease were more likely than the control group to have premorbid personality disorders [131]. Notably, studies have shown that personality traits are associated with cognitive function over time in middle-aged adults. Specifically, in one study of 510 adults (average age = 57.6 years), neuroticism, as measured on a personality inventory, was associated with age-related cognitive decline over an average time of 9 years, while higher conscientiousness appeared to minimize this effect [132]. Although not evaluating incident dementia itself, a study of 120,640 participants from nine datasets found that higher neuroticism was associated with worsened episodic memory when measured at more than two assessments and that conscientiousness was associated with less decline in memory function when measured at more than two assessment times [133]. A meta-analysis of 12 studies found that neuroticism was associated with an increased risk of dementia (hazard ratio: 1.24; 95 percent confidence interval: 1.17 to 1.31) [134]. This study also found that lower extraversion and lower agreeableness were associated with an increased risk of dementia while higher conscientiousness and higher openness were associated with a reduced risk [134]. Based on data from the Health and Retirement Study over a period of 14 years and from the English Longitudinal Study of Ageing over a period of 8 years, Stephan et al. [135] found that neuroticism was associated with the risk for incident dementia whereas agreeableness, conscientiousness, extraversion, and openness were associated with a decreased risk for subsequent dementia. In a study that evaluated associations between three scales of the Minnesota Multiphasic Personality Inventory with a median follow-up time of 20.2 years, the anxious trait was associated with incident Parkinson’s diseases, although the depressive trait was not [136]. As these findings suggest, it is possible that even personality traits may contribute to age-related neurocognitive function and that more extreme personality traits including those associated with personality disorders may contribute to the risk of dementia. While the mechanisms underlying these associations are unclear, it has been suggested that perceived stress and vulnerability to stress and anxiety may be contributing factors [137].

Findings have suggested associations between personality disorders and personality traits such as neuroticism and the risk for subsequent dementia, although additional research is needed, including studies evaluating associations between specific personality disorders and incident dementia. Future studies will need to include both personality traits as well as personality disorders in longitudinal designs to evaluate their possible contribution to the risk of dementia.

## 3. Discussion

The main finding of this narrative review is that accumulating research has associated some psychiatric diseases with incident dementia. While additional research is clearly needed, the associations between psychiatric diseases including bipolar disorder [28], depression [11], anxiety [53], ADHD [18], PTSD [64], obsessive–compulsive disorder [19], autism spectrum disorder [99], schizophrenia [17], and personality disorder [131] and personality traits [132] and incident dementia suggest the possibility that these conditions might be risk factors for the subsequent development of dementia. The number of available studies investigating the association between psychiatric disorders and incident dementia differs between individual psychiatric disorders, highlighting important gaps in research investigating these associations.

However, the available findings suggest the non-trivial nature of the associations between psychiatric disorders and incident dementia. Based on the results of their 30-year longitudinal study of associations between mental disorders and subsequent dementia, Richmond-Rakerd et al. [13] noted that the associations between mental disorders and subsequent dementia were stronger than the associations between other medical disorders and later dementia. In the studies we reviewed, effect sizes varied somewhat both within and between psychiatric disorders. Despite this variation, these effect sizes provide at least an estimate of the magnitude of the risk between psychiatric disorders and dementia. In this regard, Diniz et al. [25], for example, report an odds ratio of 2.36 for the association between bipolar disorder and frontotemporal dementia, and Huang et al. [23] and Faustino et al. [29] reported a hazards ratio of 6.78 and an odds ratio of 3.35, respectively, for associations between bipolar disorder and Parkinson’s disease. The identification of factors accounting for differences in the magnitude of risk both within and across psychiatric disorders is needed to better characterize the possible associations between psychiatric disorders and dementia.

A variety of mechanisms could account for the putative associations between psychiatric conditions and dementia. In their meta-analysis and systematic review, Stafford et al. [26] listed the possible mechanisms underlying the putative associations between psychiatric disorders and dementia including inflammation, the relationships between psychiatric disorders and cardiovascular disease, associations between psychiatric disorders and poor health behaviors, lowered cognitive reserve, and stress associated with psychiatric disorders. Further, associations between psychiatric diseases and subsequent dementia raise the possibility that clinically manifested changes associated with later dementia may become apparent decades before the onset of dementia and neurodegeneration [26]. That is, some cases of the psychiatric diseases we reviewed that were associated with subsequent dementia could be on a cognitive trajectory leading to dementia. However, Schneider and Ling [138] note in an editorial commenting on findings showing associations between traumatic brain injury, post-traumatic stress disorder, and depression in female military veterans that much more research is needed on the possible associations between psychiatric disorders and subsequent dementia and the associated mechanisms. Nonetheless, if additional findings support this hypothesis that psychiatric disorders are associated with incident dementia, then it may be that abnormalities in brain function could be present early in life even in early childhood, such as would be the case, for example, in ADHD, autism spectrum disorders, and schizophrenia. As such, early identification and intervention could mitigate the risk between these conditions and risk of dementia later in life.

A primary limitation of the research as a whole evaluating the relationship between psychiatric disorders and incident dementia is the overall lack of research addressing this question. To further evaluate the hypothesis that psychiatric disorders such as major depression, bipolar disorder, anxiety, PTSD, autism spectrum disorder, OCD, ADHD, schizophrenia and other psychosis disorders, personality disorders, and personality traits are risk factors or even precursors for neurodegenerative disease, additional large longitudinal studies evaluating the associations between psychiatric disorders and subsequent neurodegenerative disease are needed, as are additional meta-analyses evaluating reported associations between specific categories of psychiatric disorders and neurodegeneration. Further, research investigating possible mechanisms and biochemical pathways is needed. As it is likely that not all people with major depression, bipolar disorder, or attention-deficit/hyperactivity disorder subsequently develop, or are at increased risk for dementia, research investigating the reasons why some people with these conditions are more vulnerable than others to develop neurodegeneration is needed. Alternatively, there is a need to identify factors that protect against the occurrence of dementia in people with psychiatric disorders. One approach to investigating factors in psychiatric disorders associated with later dementia could be to identify transdiagnostic genetic, molecular, or environmental factors that confer risk for the development of dementia.

If the putative associations between psychiatric diseases and incident dementia are indeed valid, then a critical clinical and theoretical question concerns how treatment for psychiatric disorders could alter the subsequent risk of neurodegeneration. Lithium treatment of bipolar disorder appears to be neuroprotective [28], which suggests the possibility that the treatment of bipolar disorder with lithium could offer some protection against the later development of neurodegeneration in bipolar disorder. If lithium does alter then progression to dementia in bipolar disorder, then it will be important to identify whether lithium could modify the association between other psychiatric disorders and incident dementia. An additional important question is whether lithium is neuroprotective because it prevents extreme cycles in mood and behavior, thereby protecting the brain, or whether it is because the medication is neuroprotective against neurodegeneration. Similarly, it is also unknown whether the available treatments for psychiatric conditions, including modalities such as transcranial magnetic stimulation, pharmacotherapy, and psychotherapy, alter the association between psychiatric disorders and later neurodegeneration. In a meta-analysis of 18 longitudinal studies [43], antidepressant treatment use in depression increased the risk of subsequent dementia (risk ratio: 1.37; 95 percent confidence interval: 1.11 to 1.70) and mild cognitive impairment (risk ratio: 1.20; 95 percent confidence interval: 1.02 to 1.42) compared to people with depression who did not use antidepressants. Perhaps those treated with antidepressants in the four included studies, two of which had risk ratios for dementia (and 1 of 2 studies evaluating risk of MCI) that overlapped with 1.0, were in some ways different (e.g., more severe, differed in comorbid health conditions or other risk dementia risk factors) than those with depression that were not treated with antidepressant medications. The study with the largest sample (*n* = 406) investigating risk of dementia and antidepressant use [139] included only patients hospitalized between 1959 and 1963 for mood disorders, conducted follow-up every five years until 1985, and did not include cognitive evaluations when making the dementia diagnosis. Furthermore, the diagnosis and ability to rule out other conditions, perhaps by neuroimaging or genetic studies, was vastly different in 1985 than it is now. Additionally, antidepressant treatment available in the 1960s and 1970s was much different from now. Observational studies, which of course do not include randomization to treatment and cannot address causation, also likely do not provide enough detail to know why patients were or were not treated with antidepressant medication or why specific types of medications were chosen. Additional research is needed to better understand how treatment interventions affect the associations between the psychiatric disorders reviewed herein and the risk of subsequent dementia.

An investigation of the mechanisms underlying the associations between psychiatric disorders and subsequent dementia could offer new strategies for dementia prevention and treatment. If some psychiatric disorders are truly associated with subsequent dementia, then research will need to investigate whether psychiatric disorders, or a subset of psychiatric disorders, are potentially modifiable risk factors for dementia.

If additional research supports the hypothesis that some neuropsychiatric disorders are risk factors or precursors to neurodegenerative diseases in some cases, then there would be more reason to investigate the etiologies of the neuropsychiatric conditions associated with neurodegeneration, with the goal of both preventing these disorders from occurring and for developing additional treatments in an attempt to treat the psychiatric disorders themselves and to prevent the occurrence of dementia. Similarly, if additional research supports the association between psychiatric disorders and incident dementia, then the presence of a psychiatric disorder could alert a treating clinician to an increased risk for dementia, possibly resulting in earlier diagnoses of and treatment implementation for dementia.

While the strengths of this narrative review are its inclusion of studies evaluating the associations between nine different psychiatric disorders in addition to personality disorders and personality traits and incident dementia, and its use of data from meta-analyses where possible, it has several limitations. We may have inadvertently excluded relevant studies from our analysis. Further, we did not evaluate the quality of the studies we included in the review, possibly leading to bias from low-quality studies.

## 4. Conclusions

Based on the results of this narrative review, we conclude that the available findings provide support for the hypothesis that depression, bipolar disorder, anxiety, PTSD, OCD, ADHD, autism spectrum disorder, schizophrenia and other psychosis syndromes, and personality disorders and traits might be associated with incident dementia. The possible associations between these disorders and incident dementia suggest important theoretical and clinical implications. These associations could give insights into the causes and nature of both dementia and psychiatric disorders and provide novel means of preventing or treating dementia. Nonetheless, these findings remain tentative and sensitive to new findings. Additional research is required to better and more fully characterize the associations between psychiatric disorders and incident dementia. This research would need to use more large longitudinal studies and include meta-analytic and meta-regressive analyses, while including potentially confounding variables into the study designs. In some cases, it is unclear, particularly in late-onset psychiatric illness, if the observed psychiatric disease has clearly established itself before the onset of incident dementia or if it is simply an early manifestation of the neurodegenerative or dementing condition. Based on the findings of this narrative review, we believe that the current and projected numbers of cases of dementia and the high prevalence globally of psychiatric disorders indicate the need for further research investigating the associations between psychiatric disorders and incident dementia.

## Data Availability

Not applicable.
